# RNA Modification and Digestive Tract Tumors: A Review

**DOI:** 10.2174/0109298673350007241031025153

**Published:** 2025-01-08

**Authors:** Yafeng Liu, Shujun Zhang, Kaijie Liu, Xinyu Gu, Xinjun Hu

**Affiliations:** 1 Department of Infectious Diseases, The First Affiliated Hospital, College of Clinical Medicine, Henan University of Science and Technology, Luoyang, 471000, Henan, China;; 2 Department of Oncology, The First Affiliated Hospital, College of Clinical Medicine, Henan University of Science and Technology, Luoyang, 471000, Henan, China;; 3 Henan Medical Key Laboratory of Gastrointestinal Microecology and Hepatology, Luoyang, 471000, China

**Keywords:** RNA modification, regulatory factors, detection methods, digestive tract tumor, treatment, immunological detection

## Abstract

Gastrointestinal tumors, including colorectal and liver cancer, are among the most prevalent and lethal solid tumors. These malignancies are characterized by worsening prognoses and increasing incidence rates. Traditional therapeutic approaches often prove ineffective. Recent advancements in high-throughput sequencing and sophisticated RNA modification detection technologies have uncovered numerous RNA chemical alterations significantly associated with the pathogenesis of various diseases, notably cancer. These discoveries have opened new avenues for therapeutic intervention. This article delves into epigenetic modifications, with a particular emphasis on RNA alterations such as N6-methyladenosine (m6A), 5-methylcytosine (m5C), 1-methyladenosine (m1A), 7-methylguanosine (m7G), and N4-acetylcysteine (ac4C). It examines the functions and mechanisms of action of regulatory entities known as “Writers,” “Readers,” and “Erasers” to these modifications. Additionally, it outlines various methodologies for detecting these RNA modifications. Conventional techniques include radioactive isotope incorporation, two-dimensional thin-layer chromatography (2D-TLC), mass spectrometry, and immunological detection methods. Specialized methods such as bisulfite sequencing and reverse transcription stops are also discussed. Furthermore, the article underscores the significance of these modifications in the development, progression, and therapeutic targeting of gastrointestinal tumors, including esophageal, gastric, colorectal, liver, and pancreatic cancers. This exploration provides foundational insights for enhancing diagnostic accuracy, treatment efficacy, and prognostic assessment in gastrointestinal oncology.

## 1 INTRODUCTION

The field of epigenetics has expanded significantly in recent times. Central to this field is the study of how epigenetic mechanisms, such as chemical changes, chromatin structure reconfiguration, and the role of noncoding RNAs, influence gene expression and hereditary information transmission without changing the DNA sequence. Key components include DNA methylation, histone modifications, and non-coding RNA regulation. These factors are crucial in cell growth, differentiation, and the development and progression of diseases. Research interest has grown from focusing primarily on DNA and histone modifications to including the extensive RNA modification area. RNA modifications, such as m6A, m1A, m5C, m7G, ac4C, 2′-o-Me, A-to-I editing, and pseudo uridylation pseudo uridylation, play a key role in not only gene expression and disease development but also development and progression of a multitude of diseases. This review emphasizes the critical role of RNA modifications in gastrointestinal tumor progression, outlines the structural features of regulatory elements, and discusses the current applications of epigenetic modifications in treating these cancers. It also sets the stage for future diagnostic, therapeutic, and prognostic approaches in gastrointestinal oncology.

### 1.1 m6A Modification of RNA

Various RNA types, including mRNA, tRNA, miRNA, rRNA, circRNA, and lncRNA, are ubiquitously expressed and perform distinct functions. mRNA is particularly crucial, acting as an intermediary that facilitates protein synthesis through transcription, splicing, transportation, and translation.

m6A modifications are prevalent in different stages of protein translation and serve multiple roles. As one of the most studied and common RNA modifications, m6A represents the typical formation of N6-methyladenosine through methylation at the sixth position of adenosine. These modifications help in the recruitment of RNA-binding proteins. Although m6A can pair with uracil, m6A·U base pairs are less stable than the conventional A·U base pairs. The presence of m6A tends to disrupt the RNA's ability to form stable structures, making it more linear or unfolded, which increases accessibility to proteins that bind to single-stranded RNA.

In the nucleus, m6A is primarily found in conserved regions near the 3′ untranslated regions (UTRs) and stop codons of transcripts. The m6A modifications are introduced by “Writer” complexes, such as Methyltransferase Like 3(METTL3) and METTL14 and can be recognized by “Readers” including YTH domain-containing protein 1(YTHDC1), YTHDF1, YTHDF2, YTHDF3, Eukaryotic initiation Factor 3 (eIF3), METTL3, Heterogeneous Nuclear Ribonucleoprotein C(HNRNPC), HNRNPG, Insulin-like Growth Factor 2 mRNA Binding Protein 1(IGF2BP1), IGF2BP2, IGF2BP3, and Fragile X Mental Retardation Protein(FMRP). These modifications can be reversed by “Erasers” such as FTO and Fat Mass and Obesity-Associated protein (FTO) AlkB Homolog 5(ALKBH5), which regulate their effects. Initially, m6A was associated with increased instability, mainly regulated by YTHDF2 and METTL3 [1].

Furthermore, m6A is crucial in controlling mRNA's translational function. This process relies on the m6A reader YTHDF1, which recruits ribosome subunits and boosts translation by interacting with the translation initiation factor eIF3 [2]. This role is predominantly supported by m6A modifications near the stop codon and 3′ UTRs, although modifications at the 5′ UTRs can also enhance translation [3].

The enzymatic activity of METTL3 depends on another Writer, METTL14, with which it forms a complex. In this complex, METTL14 acts as an RNA-binding subunit, while METTL3 functions as the catalyst for m6A RNA modification.

The effect of m6A on mRNA splicing remains unclear. However, research by Wen Xiao *et al.* has shown that YTHDC1 plays a role in mRNA splicing by influencing splicing factors. Other “Readers” such as YTHDF1, YTHDF2, and YTHDF3, part of the YTH family, control mRNA degradation [4-8], translation [9-17], and stability [16, 18-22], and have varied regulatory functions. For example, YTHDF1 is involved in signal transduction [11]. and DNA damage repair [19]; YTHDF2 is linked to inflammatory pathways [23] and oncogene expression maintenance [24]; and YTHDF3 influences gene expression [25].

Zheng Guanqun and colleagues increased m6A modification on mRNA by disrupting the ALKBH5 gene, leading to significantly enhanced mRNA export from the nucleus. This finding indicates a strong connection between ALKBH5 absence and nuclear mRNA export [26]. Besides YTH domain proteins, heterogeneous nuclear ribonucleoproteins (HNRNPs), such as HNRNPG, HNRNPC, and HNRNPA2B1, also recognize m6A. These RNA-binding proteins regulate various cellular processes by attaching to single-stranded RNA-binding motifs, the discovery of which often depends on m6A modifications.

HNRNPG is synthesized alongside RNA polymerase II and plays a crucial role in modulating mRNA splicing [27]. It uses its RNA recognition motif (RRM) and Arg-Gly-Gly (RGG) sequences. HnRNPC, mainly composed of core hnRNP particles, targets uridine-rich sequences to facilitate splicing [28-30]. HnRNPA2B1 features two RRMs at its N-terminus and a glycine-rich segment at its C-terminus, including an RGG box. Its interaction with RNA, particularly through the RRM and RGG box regions, is essential for enhancing mRNA splicing efficacy and stability [31].

The IGF2BP family of mRNA-binding proteins includes IGF2BP1, IGF2BP2, and IGF2BP3, which recognize N6-methyladenosine in mRNA transcripts through a common GG(m6A)C motif. These proteins possess two RNA-recognition motifs and four K homology domains. They function in both the nucleus and the cytoplasm, with the KH domain primarily involved in RNA binding and the RRM domain enhancing the stability of the IGF2BP-RNA complex. This stability contributes to a longer mRNA lifespan, increased mRNA stability, and improved translation [32].

In ribosomal RNA, m6A modifications have been identified at two conserved sites: adenosine position 1832 in human 18S rRNA and adenosine position 4220 in human 28S rRNA. These modifications, catalyzed by Zinc finger CCHC domain-containing protein 4(ZCCHC4) for 28S rRNA and METTL5 for 18S rRNA [33], introduce structural changes in rRNA. They play a role in creating ribosomal diversity and driving specific translational responses under various conditions.

Recent studies on circular RNAs highlight their role as molecular sponges, particularly focusing on the m6A modification. This modification is typically added by METTL3 and can be recognized by YTHDC1 or Kinase Interacting with the Armadillo repeat-containing protein 1299 (KIAA129), further processed by METTL14 and Wilms tumor 1 Associated Protein (WTAP), and influenced by IGF2BP2. It can also be decoded by YTHDF3 and eIF4G2, regulated by Yes Associated Protein 1(YAP1) and YTHDF1, with ALKBH5 responsible for its removal [1, 34]. In circRNAs, the m6A modification enhances biogenesis through interactions with YTHDF3 and eIF4G2 and initiates cap-independent translation of open reading frames, as seen in circZNF609. Additionally, m6A modifications in long non-coding RNAs (lncRNAs) have been shown to promote tumorigenesis and gene silencing [1].

### 1.2 m1A Modification of RNA

m1A modifications are frequently observed in RNA molecules, especially at the first nitrogen atom of adenosine. These modifications are introduced by enzymes such as tRNA Methyltransferase 6 (TRMT6), TRMT61A, TRMT61B, TRMT10C, and Nuclear Myosin 1-like Protein (NML), and are recognized by proteins such as YTHDF1, YTHDF2, YTHDF3, and YTHDC1. They can be removed by demethylases including ALKBH1, ALKBH3, ALKBH7, and FTO. Typically found in the 5′ UTR, coding sequence, or 3′ UTR of mRNA, m1A modifications improve the efficiency of translation initiation and elongation by altering RNA structure and stability. These modifications are believed to enhance ribosome release, mRNA metabolism, translation processes, and protein production. High concentrations of m1A modifications in the mitochondrial RNA that encodes the NADH dehydrogenase subunit 5(ND5) gene hinder translation and protein synthesis within mitochondria. This occurs by impeding mitochondrial ribosome activity, leading to incorrect amino acid assembly and truncated proteins [35]. m1A modifications are common in GC-rich regions, with the sequence GUCNANNC as the primary recognition site. In these regions, T-loop or hairpin structures, which may be maintained by m1A modifications facilitated by the TRMT6/TRMT61A complex, are likely to form. Additionally, a small amount of m1A may be found in GA-rich regions. Notably, the presence of m1A near the mRNA cap in the 5′ UTR significantly enhances translation efficiency. In human mRNA, m1A is located at the translation start site, the first splicing site, and around the highly structured region of the start codon. This distribution of m1A is consistently associated with increased translation efficiency and protein levels. Furthermore, m1A levels can change dynamically in response to various stressors [36].

m1A carries a positive charge, which can disrupt Watson-Crick base pairing with uridine. This disruption affects the formation of specific mRNA structures and impacts physiological functions. m1A regulates mRNA translation in two ways: in the 5′ UTRs, it uses its positive charge to disrupt base pairing, affect RNA secondary structure formation, and promote RNA double strands' unwinding, enhancing translation initiation and efficiency. In the Coding Sequence (CDS) region of mRNA, m1A may inhibit translation by interfering with ribosome scanning mechanisms or release factor binding [37]. Additionally, the presence of m1A on transcripts is closely linked to gene expression levels, with differential m1A methylation showing a positive correlation with gene expression [38].

The formation of m1A epigenetic marks involves the TRMT6-TRMT61A protein complex [39], where TRMT61A functions as the enzymatic subunit and TRMT6 provides recognition capabilities. Research suggests a link between high TRMT6 expression and crucial cellular functions such as cell cycle progression and signaling pathways [40]. The TRMT6/TRMT61A complex influences cellular activities such as proliferation, migration, and necrosis by regulating the translation of targeted mRNAs [41]. ALKBH3, the enzyme responsible for removing m1A modifications from mRNA, uses dual β-hairpin structures and α2 helices to bind single-stranded substrates. Critical amino acid residues present within the active site pockets, such as Thr133, and in their surrounding regions are essential for m1A recognition and function. ALKBH3 operates both in the nucleus and in the cytoplasm, playing a role in controlling mRNA m1A demethylation [37, 42, 43].

Proteins such as YTHDF1, YTHDF2, YTHDF3, and YTHDC1 specialize in detecting m1A modifications on mRNA. These proteins, belonging to the YTH family, bind m1A through hydrophobic pockets that form around conserved residues in the YTH domain, such as Trp432 [44]. The interactions between these reader proteins and mRNA likely influence RNA behavior through mechanisms such as hydrogen bonding, base stacking, and RNA structural changes [45]. For example, YTHDF1 enhances translation and boosts cellular glucose metabolism by binding to m1A on mRNA; YTHDF2 helps regulate the stability of m1A-containing transcripts; and YTHDF3 affects mRNA decay [46], translation, macrophage polarization, and various aspects of cellular behavior [47].

tRNA contains m1A modifications at several positions, including 9, 14, 22, 57, and 58, with positions 9 and 58 also found in mitochondrial tRNAs. These modifications are introduced by enzymes such as TRMT6/TRMT61A, TRMT10C/ Short-Chain Dehydrogenase/Reductase Family 5C Member 1(SDR5C1), and TRMT61B, and removed by demethylases such as ALKBH1, ALKBH3, and FTO [48]. For instance, m1A57 temporarily forms as an intermediate in converting into 1-methylcytosine (m1I) *via* water deamination. The modifications at m1A9 and m1A58 help maintain the structural integrity and proper folding of tRNAs. Specifically, m1A9 helps preserve the L-shaped structure of tRNA by stabilizing unstable Watson-Crick interactions. Furthermore, m1A9 modifications impact cellular functions, and mitochondrial respiration, and play roles in regulating muscle tone and neonatal diseases. The m1A58 modification in human tRNA-Lys3 is crucial for the accuracy and efficiency of retroviral reverse transcription, including Immunodeficiency Virus type 1(HIV-1). Methyltransferases responsible for m1A modifications belong to the Resting Metabolic Rate Fat-Free Mass(RFM) or SpoU rRNA Methylase Family(SPOUT) superfamily and use S-adenosyl-L-methionine (SAM) as a methyl donor. This process links tRNA methylation to ATP and methionine production. Proteins in the RFM family feature Rossmann-fold structural motifs that bind adenosine cofactors, such as SAM and NADH [49].

In conclusion, m1A modifications primarily enhance the processing of precursor RNA in tRNA and play a key role in the regulation of translation initiation and efficiency. These modifications help recruit modified tRNAs, optimizing the translation process and ensuring the structural integrity of the tRNA T-loop and overall RNA stability [37].

### 1.3 m5C Modification of RNA

m5C modification, which involves adding a methyl group to the fifth carbon atom of the cytosine ring within DNA and RNA, follows a specific recognition sequence. In Arabidopsis thaliana, this sequence includes HACCR (where H represents A, C, or U; R signifies A or G), CTYCTYC (Y denotes C or U), CCDCCR (D stands for A, U, or G), and CWUCUUC (W indicates A or U). In murine models, m5C is mainly found downstream of the translation initiation site. Detection methods have identified m5C modifications primarily in the 5′ UTR, CDS, 3′ UTR, and near the translation initiation site. Detailed localization studies reveal an enrichment of m5C in GC-rich regions, a consistent pattern across various species [50]. At the molecular level, m5C enhances RNA structural stability through improved base stacking and increased thermal stability of hydrogen bonds with guanine [51]. The post-transcriptional modifications involving 5-methylcytosine affect mRNA maturation, stability, and translation efficiency, playing critical roles in numerous biological and pathological processes, including stress responses, oncogenesis, cancer cell migration, embryogenesis, and viral replication [52].

In mRNA, the m5C modification involves various regulatory elements and is primarily facilitated by two main types of RNA methyltransferases: DNMT2 and the NSUN protein family, which includes enzymes such as NOP2/Sun RNA methyltransferase 2 (NSUN2), NSUN4, and NSUN6. Proteins such as Y-Box Binding Protein 1 (YBX1), Aly/REF export factor (ALYREF), RAD52 DNA Recombination and Repair Protein (RAD52), Lin-28 Homolog B (LIN28B), and FMRP, which act as 'readers', recognize this modification. Meanwhile, “erasers” such as ALKBH1 and Ten Eleven Translocation Methylcytosine Dioxygenase 1 (TET1) remove m5C modifications. These elements play vital roles in numerous cellular processes including mRNA stabilization, translation enhancement, nuclear exportation, adipogenesis inhibition, glycolysis enhancement, exosome secretion, tumor aggressiveness, chemoresistance, and signal transduction. They are crucial for cell proliferation, invasiveness, and cell cycle control [53]. The methylation activity of NSUN proteins depends on the functionality of S-adenosylmethionine (SAM), which features an RNA recognition motif (RRM) and a Rossman-fold catalytic core for accommodating SAM cofactors. NSUN proteins operate using two catalytic cysteine residues, whereas DNA methyltransferase 2(DNMT2) functions as a DNA methyltransferase with a single active site containing a half-cystine residue. The m5C formation mechanism involves creating covalent intermediates between cysteine and cytosine within RNA, which activates the electron-deficient pyrimidine heterocyclic ring, facilitating a nucleophilic attack on the SAM methyl group [51]. NSUN1, NSUN2, and NSUN5 are found across eukaryotes, while other NSUN protein family members are specific to more complex eukaryotic organisms.

In tRNAs, the m5C modification is vital for enhancing codon-anticodon pairing, maintaining homeostasis, responding to stress, and ensuring efficient and precise translation. Key regulatory enzymes include DNMT2, NSUN2/3/6, TET2, and ALKBH1. Specifically, NSUN6 and DNMT2 target C72 and C38 sites in certain tRNAs, respectively. NSUN2 has a broader target range, affecting multiple sites (C34, C40, C48, C49, and C50) in various tRNAs and other RNA substrates. While tRNA modifications can occur at different stages of biosynthesis, most enzymes responsible are in the nucleus, suggesting that most modifications occur early in biosynthesis. NSUN2, primarily found in the nucleus, modifies the C34 site on TRNA-LEU (CAA) in intron-containing precursors. DNMT2, located in both the nucleus and cytoplasm, suggests that m5C modifications may also occur later in tRNA biosynthesis. In contrast, NSUN6 is located in the cytoplasm, near the Golgi apparatus and centrioles matrix, indicating that C72 methylation occurs during the later stages of maturation after nuclear export.

Modifications of tRNA can influence protein translation by affecting how codons and anticodons interact. For example, increased m5C methylation at the C34 site of tRNA-Leu-(CCA) in yeast boosts translation during oxidative stress. Methylation at the C38 site in the tRNA-Asp-(GUC) anticodon loop by DNMT2 also enhances the translation of certain gene groups. This modification by Dnmt2 not only improves translation fidelity but may also help the cell respond to stress. Furthermore, m5C modifications outside the anticodon loop can alter the structural integrity of tRNA. Modifications at M5C-48/49/50 within the variable loop at the T-stem junction, dependent on NSUN2, are an example. The interaction between C48 and G15 in the D-loop, known as the “Levitt pair,” is crucial for the tRNA to form its characteristic L-shaped tertiary structure. The presence of m5C at site 48 improves base stacking, thus strengthening this interaction and stabilizing the tRNA structure [51].

NSUN3 assists in adding m5C at the C34 site on mt-tRNA-Met, a key step for the function of dioxygenase ATP-binding Cassette Subfamily H Member 1 (ABH1) in human and mouse mitochondria. The cooperation between NSUN3 and ABH1 at this site enhances codon recognition during mitochondrial translation. NSUN3's role is vital for mitochondrial function, affecting both respiratory activity and protein synthesis [50].

In ribosomal RNA, the modification known as m5C plays a role in modulating bacterial resistance and maintaining the stability of the rRNA-tRNA-mRNA complex under stress. Key regulatory proteins include NSUN1/4/5 and YTHDF2. Eukaryotic ribosomal RNAs feature a variety of base modifications, notably two m5C sites: position 3761 in human/2870 in yeast and position 4413 in human/2278 in yeast on the 28S/25S rRNA. In humans and yeast, the NSUN5/Rcm1 protein methylates the 28S C3761/25S C2278 site, while the NSUN1/Nop2 protein targets the 28S C4413/25S C2870 site. These modifications are located near the peptidyl transferase center and the eB14 intersubunit bridge in the large subunit of the mature ribosome, suggesting that m5C modifications on the 25S/28S rRNA may help regulate intracellular translational processes. Schosserer *et al.* confirmed that the modification pattern mediated by Replication Component 1(Rcm1)/NSUN5 enhances stress resistance and life extension. NSUN4 plays a crucial role in ribosome assembly and translation within the large ribosomal subunit [50]. Since m5C enhances RNA structural stability, its presence in rRNA likely supports the proper folding of rRNA in essential functional areas of the ribosome [51], thus playing a unique role in modification.

### 1.4 m7G Modification of RNA

m7G is a nucleotide modification that involves the addition of a methyl group to the N7 position of guanine, typically using SAM as the methyl donor. This modification was initially discovered at the 5′ end of mRNA in eukaryotic cells. The RNA guanine-7 methyltransferase(RNMT)/ RNA guanine-7 methyltransferase methyltransferases-associated protein (RAM) methyltransferase complex uses SAM to create the m7G(5′) pp (5′)X cap structure, often referred to as the “hat” structure, at the N7 site of the guanine nucleotide.

Advancements in detection methods have revealed m7G modifications not only in the 5′ UTR, CDS, and 3′ UTR of mRNA but also predominantly in AG-rich regions. In the nucleus, the m7G cap structure is identified by a cap-binding complex (CBC) composed of CBP80 and CBP20, which is crucial for its functionality. These functions include enhancing mRNA stability, aiding ribosomal engagement, participating in splicing events, regulating both transcription and translation initiation, and contributing to nonsense-mediated mRNA decay (NMD).

After being moved to the cytoplasm, mRNAs with the m7G cap attach to the eukaryotic translation initiation factor (eIF4E), playing a role in the control of translation initiation. The methylated cap structure enhances mRNA nuclear export by attracting Nucleolar Protein Like 3 (Npl3) and RNA Binding Protein Yra1 (Yral) proteins, and also improves RNA stability by inhibiting RNA hydrolase activity. Although m7G is a common element in mRNA, during heat shock and oxidative stress, the levels of m7G modifications in the mRNA CDS and the 3′ UTR increase significantly. In contrast, m7G levels in the 5′ UTR decrease markedly. This change leads to more efficient mRNA translation. Given the critical roles of the 3′ UTR in gene expression and translation regulation, and the CDS in encoding proteins, it appears that increased m7G modifications in the CDS and 3′ UTR are associated with higher protein production, while reduced modifications in the 5′ UTR are linked to lower protein production.

Similar to m6A modification, m7G modification plays a crucial role in the regulation of mRNA nuclear export and and translation and protects RNA from exonucleolytic degradation. This modification is regulated by various factors, including “writer” elements such as RNMT/RAM, METTL1/ WD repeat-containing protein 4(WDR4), and Williams-Beuren syndrome chromosome region 22(WBSCR22)/TRMT112, as well as “reader” components from the eIF4E family (eIF4E1/eIF4E, eIF4E2/4EHP, eIF4E3), and Ago2. These factors primarily affect mRNA stability by interacting with the cap structure of the mRNA 5′ UTR, which influences its nuclear export and translation dynamics [48]. METTL1, a key RNA m7G methyltransferase, typically forms a complex with WDR4 to catalyze m7G modifications at the G46 site within the tRNA variable loop, enhancing tRNA stability and affecting its structure and function. In Saccharomyces cerevisiae, m7G is involved in the rapid decay pathway of tRNA, regulating tRNA levels. In thermophiles, m7G modification in precursor tRNA enhances the effectiveness of other tRNA-modifying enzymes. The METTL1/WDR4 complex also affects mRNA translational efficiency through changes in tRNA m7G modification and is linked to neurological disorders such as Alzheimer's disease. Reduced METTL1 expression leads to decreased ribosomal accumulation and translocation at the tRNA's A-site. Conversely, increased METTL1 expression boosts m7G tRNA methylation (notably Arg-TCT tRNA), reduces ribosomal stalling at AGA codons, and promotes cell cycle progression. WDR4 is essential for METTL1's enzymatic activity and is associated with conditions involving growth retardation, craniofacial anomalies, and seizures. RAM, as a subunit activator for RNMT, helps recruit methyl donors within the complex and enhances RNMT's methyltransferase activity, thus affecting m7G modification and gene expression related to the mRNA cap region.

Contemporary research focuses on the m7G modification in tRNA, specifically the methylation of the 46th N7 guanine atom within the variable loop. This process is primarily facilitated by tRNA methyltransferase, leading to the formation of m7G (46). This modification introduces a positive charge to the m7G (46) C13-G22 complex through hydrogen bonding with the C13-G22 base pair in the L-type tRNA conformation, thus enhancing the three-dimensional structural integrity of tRNA. Initially identified in yeast, the m7G modification is commonly found in AG sequences. m7G modification in the tRNA’s anti-codon region is vital for translational regulation, improving the translation efficiency of oncogenes and thereby accelerating tumor progression. Methylation at the G1575 site of yeast 18S rRNA is performed by the Bud23-Trm112 methyltransferase complex. A similar modification structure exists at the G1639 site of human 18S rRNA, mediated by the Bud23/Trm112 complex in yeast and the WBSCR22/TRMT112 complex in humans.

In the G1405 locus of bacterial 16S rRNA, the presence of m7G modification correlates with increased resistance to aminoglycoside antibiotics. The WBSCR22/TRMT112 complex acts as a functional counterpart to Bud23-Trm112, playing a crucial role in the m7G methylation of 18S rRNA. TRMT112, a small but universally conserved protein, is vital as an essential cofactor for WBSCR22. It is critical for both the m7G modification of rRNA and the metabolic stability of WBSCR22. Together, they form a heterodimeric methyltransferase complex that aids in the maturation of precursor rRNA into functional 18S rRNA. Besides its role in rRNA processing, WBSCR22 is involved in a variety of biological processes, including organ regeneration, wound repair, enhancement of glucocorticoid receptor activity, modulation of pulmonary inflammatory responses, and has been linked to oncogenesis and the development of chemoresistance [54].

### 1.5 ac4C Modification of RNA

The investigation revealed that ac4C modification primarily occurs within the coding sequence and the 5′ UTR of mRNA. This acetylation in the coding area significantly strengthens the structural integrity of mRNA, enhancing translation efficiency. Additionally, acetylation at the start of the 5′ UTR may influence protein synthesis by altering the arrangement of specific bases and their interactions with anticodons. Conditions, ac4C can enable translation from non-standard start sites and suppress the activity of standard start codons. The effect of ac4C on mRNA translation depends on its location; specifically, ac4C modification at the 5′ UTR Kozak sequence leads to structural changes that interfere with tRNA-iMet interactions, resulting in competitive inhibition of translation initiation. The N-acetyltransferase 10(NAT10) protein, which is crucial for ac4C modification, consists of three essential functional domains: the n-acetyltransferase domain, the ATP/GTP binding domain, and the ATPase activity domain. NAT10 also has a nuclear localization signal sequence, which specifies its location within the cell [55].

ac4C plays a critical role in various RNAs, particularly in modifying the functions of tRNA, rRNA, and mRNA. In rRNA, modifying specific bases affects not only the fidelity of RNA translation but also the dynamics of ribosome assembly. The degree of acetylation is directly linked to cellular growth rates and rRNA stability. This modification creates binding sites on rRNA for tRNA, enhancing its ability to recognize codons accurately and promoting peptide chain elongation. In mRNA, ac4C modification increases its half-life, potentially leading to the overexpression of certain genes. In yeast tRNA, N4-acetylcysteine stabilizes the internal structure at the ribose C3′ end, improves GC base pairing, and ensures precise codon recognition during translation (Table **1**) [55, 56].

## 2 TECHNIQUES FOR DETECTING RNA MODIFICATIONS

There are numerous techniques for detecting RNA modifications, and this discussion aims to encapsulate several primary methodologies. These include both general detection methods and techniques tailored for specific modifications. General detection approaches encompass radioactive isotope incorporation, two-dimensional thin-layer chromatography, mass spectrometry, and immunological detection methods. Meanwhile, specific modification detection techniques comprise bisulfite sequencing and reverse transcription stops.

Radioactive isotope incorporation stands as one of the pioneering techniques in this field. It involves pre-labeling synthetic substrates, such as nucleotides, to facilitate the modification reaction, which integrates the radioactive element with the target substrate. The quantification of the target substrate is achieved by assessing the radioactive element's content. This method is efficacious for identifying modifications like m1A, m6A, m5c, m7G, and ac4C. However, it requires prior knowledge of the target sequence's context and is restricted to *in vitro* RNA analysis.

Following this, two-dimensional thin-layer chromatography represents an enhancement over traditional thin-layer chromatography. It leverages the differential charge and hydrophobic properties between modified and unmodified RNA to effectuate their separation post-isotope or UV labeling. This technique detecting the five modifications: m1A, m6A, m5c, m7G, and ac4C. Nevertheless, its primary drawback is that it only yields methylation status information within a broad transcriptomic scope.

Additionally, several methodologies are employed to detect these nucleotide modifications, including mass spectrometry and antibody-based techniques. Mass spectrometry discerns nucleotides by comparing their mass-to-charge ratios with those of known standards. This method is akin to chromatography but does not necessitate the specific labeling of target nucleotides. Conversely, the antibody-based sequencing approach primarily utilizes the ‘methylated RNA immunoprecipitation sequencing’(MeRIP-seq) technique. This method leverages antibodies that are specific to modifications, initially developed for detecting m6A modifications. It involves eluting methylated RNA fragments following their reaction with an m6A-specific antibody. However, the efficacy of this technique is constrained by the availability and specificity of antibodies; currently, only antibodies specific to m1A, m6A, and m5C are accessible. Further development and validation are imperative for antibodies targeting m7G and ac4C modifications [57].

Bisulfite sequencing technology is particularly adept at detecting m5C. This method involves treating RNA samples with bisulfite, which converts unmodified cytosine (C) to uracil (U), while m5C-modified cytosine remains unchanged. Subsequent PCR amplification and detection enable precise determination of the position and abundance of m5C [58].

In terms of detecting m7G and m5C, the reverse transcription stops method proves more effective. This technique capitalizes on the termination effect induced by m7G and m5C modifications during reverse transcription. The process commences with the extraction of target RNA, followed by reverse transcription. Analysis of the resultant fragments from reverse transcription facilitates the determination of both the relative position and abundance of the modifications [54, 59, 60].

## 3 RNA MODIFICATION AND DIGESTIVE TRACT TUMORS

Research into RNA modification is increasingly highlighting its role in the development and progression of cancers. Studies primarily focus on the effects of RNA modifications and their regulatory factors on tumor cells, exploring the pathways through which these modifications could affect cellular functions. This insight provides a vital target for cancer therapy and lays a strong foundation for pharmaceutical development. In China, 41.6% of new malignant tumors originate in the digestive system, which also accounts for 49.3% of cancer-related deaths. Notably, more than 40% of new global malignant tumors are cancers of the esophagus, nasopharynx, liver, and stomach. Colorectal and stomach cancers are the third and fifth most common types of tumors worldwide, respectively. Lung cancer remains the top cause of cancer death, followed by colorectal, liver, female breast, and stomach cancers. Among women, colorectal cancer is the leading cause of mortality from digestive tract tumors, while liver cancer is the deadliest for men [61]. We will next explore in greater detail the critical role of RNA modification in various types of digestive tract tumors (Table **2**).

### 3.1 RNA Modification and Esophageal Cancer

Esophageal cancer is the 11^th^ most common cancer worldwide and ranks as the 7^th^ leading cause of death, with approximately 511,000 new cases and 445,000 deaths in 2022. The disease is especially prevalent in Eastern Europe and East Africa, and shows significant geographical differences, primarily between its two main types: squamous cell carcinoma and adenocarcinoma. In regions with high human development indexes, risk factors for squamous cell carcinoma include smoking and alcohol consumption, while the incidence of adenocarcinoma has been increasing yearly [61].

**Fig. (1):**
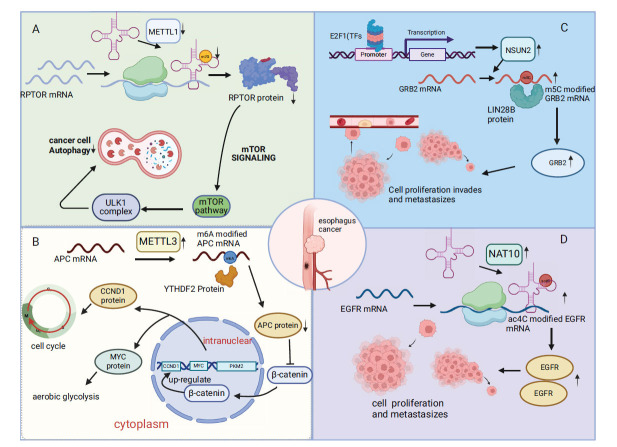
Describes the effects of m7G, m6A, m5C, and ac4C modifications on esophageal cancer. (**A**) The downregulation of METTL1 reduces the m7G modification of tRNA, resulting in a decrease in the abundance of tRNA, which leads to a decrease in the expression of decoded and translated mRNA, that is, leads to a decrease in the translation of RPTOR protein, and reduces the apoptosis induced by mTOR signaling pathway. (**B**) METTL3 and YTHDF2 jointly regulate m6A on APC mRNA to affect its protein translation and then regulate the translation of MYC and CCND1 through β-catenin to affect glycolysis and cell cycle. (**C**) E2F1 transcription factor up-regulates the expression of NSUN2, which co-regulates the m5C modification of GRB2 mRNA with LIN28B protein to promote its translation, thereby regulating cell proliferation, invasion, and metastasis. (**D**) NAT10 up-regulates the ac4C modification of tRNA to increase its abundance and promote the expression and translation of EGFR mRNA, thereby promoting cell proliferation and metastasis.

Various RNA modifications such as m7G, m5C, m6A, and ac4C play critical roles in the development of esophageal cancer. Hui Han and colleagues initially found that the m7G modification in tRNA, mediated by METTL1, is essential for the self-renewal and differentiation of mouse embryonic stem cells and also helps yeast adapt under stress conditions (Fig. **1A**). Further research revealed that METTL1 and WDR4, enzymes involved in m7G modification, are significantly overexpressed in esophageal squamous cell carcinoma tissues. Reducing m7G levels decreases the translation of certain cancer-promoting transcripts in the regulatory associated protein of mTOR complex 1 (RPTOR)/ unc-51 like autophagy activating kinase 1(ULK1)/autophagy pathway. Thus, m7G modifications in tRNA and mRNA translation influence the mTOR signaling pathway's control over autophagy in esophageal cancer cells [62, 63].

m6A, known as the most common and significant type of RNA modification, plays a crucial role in esophageal cancer. METTL3 increases m6A modification of the adenomatous polyposis coli (APC) mRNA, which leads to its degradation. This decrease in APC levels results in elevated β-catenin, which activates transcription of genes like G1/S-specific cyclin-D1(cyclin D1), avian myelocytomatosis virus oncogene cellular homolog (c-Myc), and pyruvate kinase isozymes M2 (PKM2), thereby promoting aerobic glycolysis, proliferation of esophageal squamous cell carcinoma (ESCC) cells, and tumor growth in mice (Fig. **1B**) [64]. IGF2BP1 acts as an m6A reader, recognizing and binding to mitogen-activated protein kinase kinase 6 (MKK6) and mitogen-activated protein kinase 14 (MAPK14) mRNAs in an m6A-dependent manner. This facilitates the translation of essential proteins through the p38 MAPK pathway, which plays a key role in esophageal cancer metastasis and proliferation [65]. Additionally, METTL3, FTO, ALKBH5, and IGF2BP2 are overexpressed in patients with esophageal cancer and closely linked to myc mRNA expression. Particularly, METTL3 and IGF2BP2 affect the stability and half-life of myc mRNA. METTL3 correlates positively with myc mRNA expression, contributing to the malignant progression and immune evasion in esophageal cancer. Furthermore, the study highlights the involvement of serine hydroxy methyltransferase 2 (SHMT2) in esophageal cancer development through m6A methylation [66]. Other research suggests that the WNT and Notch signaling pathways also play roles in esophageal cancer's aggressive traits [67, 68]. Interferon-induced protein with tetratricopeptide repeats 2 (IFIT2) is linked to the growth, migration, and invasion of ESCC cells and is regulated by METTL3 [69]. Additional studies indicate that the long non-coding RNA LINC0002 binds directly to the p21 protein, promoting its degradation and thus enhancing cell cycle progression and proliferation. Meanwhile, the FTO/YTHDF2 axis controls the downregulation of LINC00022, thereby influencing proliferation and tumor growth in esophageal cancer [70].

The m5C modification is implicated in various cancers, including esophageal cancer. Studies on esophageal cancer transcriptomics have shown high levels of m5C modification, associated with poor prognosis. NSUN2 induces m5C modification on the growth factor receptor-binding protein 2 (GRB2), which stabilizes its mRNA. Additionally, LIN28B contributes to the stabilization of GRB2 mRNA through signaling through the phosphoinositide 3-kinase (PI3K)/ protein kinase B (AKT) and extracellular signal-regulated kinase (ERK)/MAPK pathways. (Fig. **1C**) [71].

ac4C modification, a novel RNA modification technique, plays a regulatory role in esophageal cancer. Elevated levels of ac4C RNA modification have been observed in esophageal cancer and are strongly linked to poor prognosis. The mechanism involves reducing tRNA abundance by altering its ac4C modification, which affects mRNA decoding and translation. NAT10, the principal regulator of ac4C, is crucial. Studies have experimentally confirmed that the impact of ac4C in esophageal cancer could influence the development and progression of the disease by targeting epidermal growth factor receptor (EGFR) (Fig. **1D**) [72].

### 3.2 RNA Modification and Gastric Cancer

In 2022, there were more than 968,000 new cases of gastric cancer worldwide, with nearly 660,000 deaths. This ranks it fifth in global cancer incidence and mortality rates. Various RNA modifications, including m7G, m6A, m5C, and ac4C, have been extensively studied in gastric cancer. These modifications are key regulators of metastasis, invasion, and proliferation, and are vital for patient prognosis.

Regarding m7G modification, relatively few studies have been conducted in gastric cancer, primarily focusing on developing prognostic models. Long non-coding RNAs linked to m7G play a vital role in the tumor immune microenvironment of gastric cancer.

**Fig. (2):**
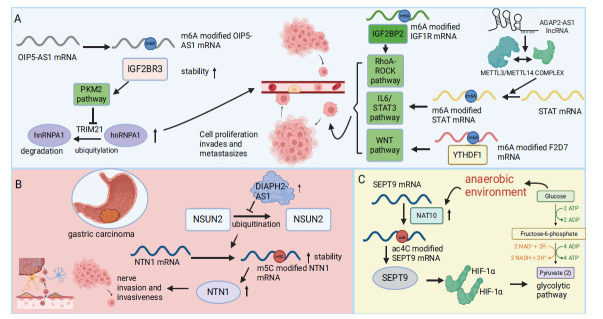
Depicts the regulatory role of m6A, m5C, and ac4C modifications in gastric cancer. (**A**) Describes four pathways that regulate the effects of cell proliferation, invasion, and metastasis. IGF2BR3 recognizes m6A on OIP5-AS1 mRNA and stabilizes its mRNA, thereby increasing its expression, and inhibits the ubiquitination degradation of hnRNPA1 through the PKM2 pathway, thereby increasing the content of hnRNPA1 and thus promoting the cell effect. Second IGF2BR2 recognizes m6A on IGF1R mRNA, promotes its expression, and promotes cell effect through the RhoA-ROCK pathway. After AGAP2-AS1 lncRNA promoted the formation of METTL3/METTL14 complex, m6A modification on STAT mRNA promoted its expression and promoted the cell effect by activating IL6/STAT3 pathway. By recognizing m6A on F2D7 mRNA to promote its expression, YTHDF1 activates the WNT pathway and thus promotes the cell effect. (**B**) Neuberger 2-AS1 up-regulation inhibits ubiquitination degradation of NSUN2, resulting in increased NSUN2 content, and promotes m5C modification of NTN1 mRNA, thereby promoting infiltration and neuroinvasion. (**C**) Hypoxia stimulates glycolysis positive feedback loop, that is, under hypoxia environment, promotes the expression of NAT10, increases the ac4C modification of SEPT9 mRNA, causes the increase of IGF2BR3 expression, promotes the glycolysis pathway through HIF-1α, and thus strengthens the hypoxia environment. Formation of anoxic /NAT10/SEPT9/HIF-1α/ glycolytic ring.

Conversely, m6A modifications exhibit various functions in gastric cancer, including enhancing metastasis, proliferation, and epithelial-mesenchymal transition. These functions are associated with regulatory elements such as ALKBH5 and IGF2BP3. The diminished expression of ALKBH5 in gastric cancer is correlated with increased tumor metastasis and lymph node involvement. Interfering with ALKBH5 expression in gastric cancer cells facilitated metastasis by upregulating Protein kinase, membrane-associated tyrosine/threonine 1(PKMYT1). IGF2BP3, acting as an m6A reader, recognized an m6A modification on PKMYT1 mRNA, enhancing its stability and promoting the invasion and migration of gastric cancer cells [73]. Additionally, IGF2BP3 interacted with opioid-induced protein 5 antisense RNA 1(OIP5-AS1), stabilizing it by recognizing its m6A modification site. This stabilization prevented the ubiquitination and degradation of tripartite motif-containing protein 21(TRIM21)-mediated heterogeneous nuclear nucleoprotein A1 (hnRNPA1) through the PKM2 signaling pathway, thereby supporting the progression of gastric cancer [74]. The interaction between tumor cells and stromal cells within the tumor microenvironment is crucial for tumor progression. Mesenchymal stem cells (MSCs), components of this microenvironment, have been increasingly recognized for their role in enhancing cancer progression through metabolic reprogramming induced by colony-stimulating factor 2 (CSF2) produced through Notch1 ubiquitination. The stability of CSF2 mRNA depends on the m6A reader IGF2BP2, which is highly expressed in gastric cancer tissues [75]. IGF2BP2 activates the RhoA-ROCK pathway by recognizing the m6A methylation site on insulin-like growth factor 1 receptor(IGF1R) mRNA, enhancing the proliferation, migration, and invasion of gastric cancer cells while reducing their tendency to undergo apoptosis [76]. The WNT pathway, crucial in gastric cancer development, is influenced by m6A epigenetic changes (Fig. **2A**). YTHDF1, by recognizing m6A sites, boosts the translation of frizzled7 (FZD7), a key Wnt receptor, leading to overexpression of YTHDF1 and increased FZD7 levels. This activates the Wnt/β-catenin pathway, promoting the progression of gastric cancer [77]. METTL3, another key factor, is vital in this context; its activation through acetylation increases its transcription and the m6A modification of hepatoma-derived growth factor (HDGF) mRNA. IGF2BP3 interacts with m6A sites on mRNA, enhancing mRNA stability and supporting tumor angiogenesis and glycolysis, thus aiding the growth and liver metastasis of gastric cancer [78]. Additionally, METTL3 enhances the m6A modification of regulation of the nuclear pre-mRNA domain containing 1B (RPRD1B) and its expression, further activating the c-Jun/c-Fos/SREBP1 axis, which increases fatty acid uptake and synthesis. RPRD1B expression is also notably increased in lymph node metastases of gastric cancer [79]. METTL3 is crucial for epithelial-mesenchymal transition (EMT) and tumor metastasis, linking high METTL3 levels in gastric cancer cells to poor prognosis. A key downstream target, zinc finger MYM-type containing 1(ZMYM1), promotes EMT and metastasis by suppressing the E-cadherin promoter *via* the C-terminal binding protein (CtBP)/ lysine-specific demethylase 1 (LSD1)/ corepressor for element-1-silencing transcription factor (CoREST) complex [80]. Additionally, METTL3 increases the m6A modification of PBX1 mRNA, enhancing its stability, which, in turn, upregulates GCH1 expression and Tetrahydrobiopterin(BH4) levels in gastric cancer cells, promoting tumor growth and metastasis [81].

In recent years, the role of circRNA modifications in gastric cancer has gained attention, especially their ability to regulate miRNA function *via* sponging mechanisms. Lowered METTL14 expression correlates with worse outcomes in patients with gastric cancer. Reduced METTL14 increases circORC5 expression and lowers Akt Substrate 1S1 (AKT1S1) and Eukaryotic Translation Initiation Factor 4B (EIF4B) levels by decreasing m6A modification on circORC5, thereby accelerating gastric cancer progression. However, this effect can be countered by miR30c-2-3p sponging through increased circORC5, which helps slow disease progression [82]. Moreover, the long non-coding RNA (lncRNA) Arf GTPase Activating Protein 2 Antisense RNA 1(AGAP2-AS1) enhances the assembly of the WTAP/METTL3/METTL14 m6A methyltransferase complex, stabilizing Signal Transducer and Activator of Transcription 3(STAT3) mRNA and activating the Interleukin 6(IL6)/STAT3 signaling pathway, which promotes cancer cell proliferation and migration [83]. Additionally, the role of FTO as an m6A demethylase in gastric cancer has been explored, showing higher FTO levels in tissues, particularly in cases with liver metastases. FTO affects the proliferation, migration, and invasion of gastric cancer cells by inhibiting caveolin-1, a protein that also regulates mitochondrial dynamics and metabolism in these cells [84].

RNA m5C modification plays a critical role in the development of gastric cancer. Studies show that during the peritoneal metastasis of gastric cancer, NSUN2 expression significantly increases, correlating strongly with metastatic advancement. The involvement of ORAI Calcium Release-Activated Calcium Modulator 2(ORAI2) is crucial in this process. NSUN2 boosts ORAI2 mRNA stability through m5C modification and enhances ORAI2 expression *via* YBX1 recognition, promoting peritoneal metastasis and the spread of gastric cancer. Furthermore, NSUN2 expression is closely linked to the activation of the AMPK signaling pathway in lipid-rich environments, enhancing Transcription factor E2F1 cis-acting element regulation [85]. Neurological invasion (NI) of gastric cancer is critically important and strongly associated with high expression of lncRNA DIAPH2 Antisense RNA 1(DIAPH2-AS1), indicating poor survival outcomes for patients. NSUN2 also increases Netrin 1(NTN1) expression and stabilizes NTN1 mRNA by enhancing m5C modification, affecting gastric cancer cell migration and NI potential (Fig. **2B**) [86]. Additionally, the expression level of lncRNA is vital for predicting gastric cancer stages. Forkhead Box C2 Antisense RNA 1(FOXC2-AS1) expression is elevated in gastric cancer tissues and cells, with higher levels indicating more advanced TNM stages and lower overall survival. The stability of FOXC2 mRNA depends on m5C methylation by NSUN2, followed by YBX1 recognition [87]. Zhang Qiang *et al.* emphasize that m5C modification adds to the complexity and variability of the gastric cancer tumor microenvironment.

ac4C modification plays a crucial role in cancer therapy by enhancing mRNA stability and translation. In gastric cancer, both ac4C mRNA modification and its regulator, NAT10, are elevated, correlating with disease progression and poor prognosis. NAT10 promotes G2/M phase progression, cellular proliferation, and tumorigenicity through ac4C modification. It also mediates acetylation of Mouse Double Minute 2(MDM2) mRNA through ac4C, stabilizing MDM2 mRNA. This leads to increased MDM2 levels and reduced p53 levels, promoting gastric cancer development [88]. Additionally, NAT10-mediated acetylation enhances the hypoxia tolerance of gastric cancer cells by increasing their glycolytic dependency (Fig. **2C**). Ac4C modification of Septin 9 mRNA activates the Hypoxia Inducible Factor-1(HIF-1) pathway and glucose metabolic reprogramming, sustaining this glycolytic dependency [89]. Neutrophil extracellular traps (NETs), released by neutrophils in response to infection or other stimuli, are made of chromatin fibers and proteins that form a network structure. Research by Donghui Liu *et al.* shows that NET levels are higher in patients with gastric cancer, especially those with metastatic disease. The interaction between gastric cancer cells and NETs, which boosts cell viability, migration, and invasion, is influenced by NAT10 and Set and MYND domain containing 2(SMYD2). Disrupting this interaction by deactivating NAT10 or SMYD2 could be beneficial. The stability of SMYD2 mRNA depends on ac4C acetylation modification by NAT10 [90].

### 3.3 RNA Modification and Colorectal Cancer

Colorectal cancer (CRC) is the third most common cancer worldwide and the second leading cause of cancer-related deaths. Societal advancements and lifestyle changes have led to an increased incidence of this disease. Studies show that men are approximately ten times more likely to develop colorectal cancer than women. High rates are observed in regions such as Europe, Australia/New Zealand, and North America, with the highest incidences reported in Denmark and Norway for men and women, respectively [61]. RNA modifications, including m1A, m6A, m5C, ac4C, and m7G, play significant roles in various cancers. In CRC, these modifications promote tumor growth, metastasis, angiogenesis, and immune suppression through various mechanisms.

Among the RNA modifications, m6A is particularly significant and widely studied. The regulatory protein METTL3 is crucial in CRC, with many studies linking its high expression to the disease's progression through processes such as angiogenesis and increased glucose uptake. METTL3 enhances CRC progression by affecting the expression of Glucose Transporter Type 1, increasing glucose uptake, and activating the mammalian target of rapamycin complex 1 (MTORC1) signaling pathway, which is essential for biosynthesis. The MTase domain of METTL3 is critical for cancer growth, with key motifs (DPPW, IHM, RTGRTGH) that are central to its carcinogenic effects; DPPW acts as a catalytic motif, while RTGRTGH is crucial for the MTase activity of the METTL3-METTL14 complex [91]. METTL3 also promotes tumor angiogenesis by modifying Ephrin-A receptor 2(EphA2) and vascular endothelial growth factor A (VEGFA) mRNAs with m6A, facilitating their recognition by IGF2BP2/3, stabilizing the mRNA, and enhancing protein translation. It further regulates the expression of vascular endothelial-cadherin (vimentin) through the PI3K/AKT/mTOR and Extracellular signal-regulated kinase 1 and 2(ERK1/2) pathways, promoting tumor angiogenesis [92].

Recent studies have highlighted the significant roles of long non-coding RNAs (lncRNAs) in regulating various cancers, including CRC. Research by Pingfu Hou and colleagues has shown that LINC00460 expression is notably higher in CRC and is associated with a poorer survival prognosis. LINC00460 promotes epithelial-mesenchymal transition and increases tumor cell proliferation, migration, and invasion. This is achieved through the interaction of high-mobility group AT-hook 1 (HMGA1) with IGF2BP2 and DEAH-box protein 9(DHX9), which stabilizes and increases the expression of HMGA1 mRNA. Additionally, METTL3-induced m6A modification affects HMGA1 mRNA expression [93]. Another lncRNA, Growth Arrest Specific 5(GAS5), impacts CRC by controlling the degradation of Yes-associated protein (YAP), a key player in signal transduction that affects cell proliferation, growth, differentiation, and apoptosis. GAS5 hinders CRC progression by interacting with YAP's WW domain in the nucleus, promoting YAP's movement to the cytoplasm and its subsequent degradation *via* ubiquitination. In contrast, YTHDF3, which is overexpressed in patients with CRC, promotes the degradation of lncRNA GAS5 by binding to its m6A modifications. This prevents the degradation of YAP, thereby activating it and contributing to CRC progression [94].

**Fig. (3):**
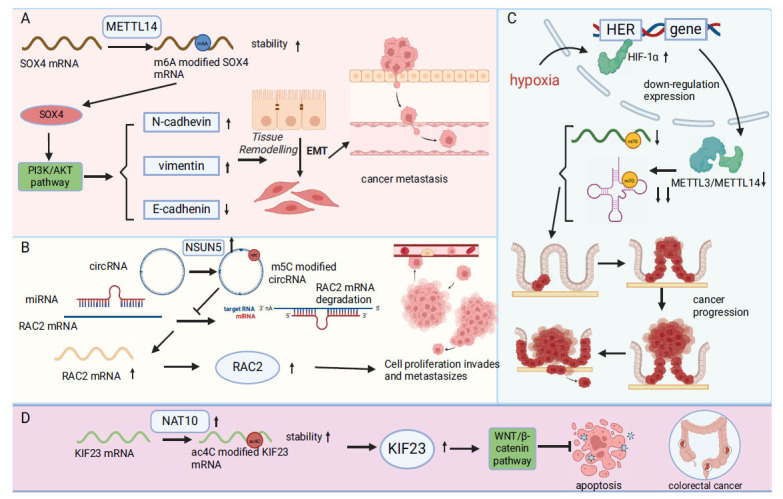
Depicts the role of m6A, m5C, m7G, and ac4C modifications in colorectal cancer. (**A**) METTL14 improves the stability of SOX4 mRNA and promotes its expression by increasing the m6A modification on SOX4 mrna. Furthermore, PI3K/AKT signaling pathway is activated to promote the expression of N-cadhevin and vimentin, while the expression of E-cadhenin is decreased, which leads to the transformation of histological types and promotes the metastasis of cancer. (**B**) The up regulation of NSUN5 increased the m5C modification of circRNA, promoted the adsorption of miRNA, thus reduced the degradation of RAC2 mRNA by miRNA, promoted the expression of RAC2, and caused cell proliferation, infiltration and metastasis. (**C**) Hypoxia conditions stimulate the up-regulation of HIF-1α, promote its binding to HER in the nucleus, and then cause the downregulation of METTL3/METTL14 complex, resulting in the reduction of m7G modification of mRNA and tRNA, which promotes the progression of cancer. (**D**) The up-regulation of NAT10 promoted the ac4C modification of KIF23 mRNA, increasing its expression, and inhibited apoptosis through the WNT/β-catenin pathway.

Distant metastasis is a major cause of death in CRC. Studies show that reduced expression of METTL14 is closely linked to metastasis, leading to poorer outcomes in patients with CRC. Lower levels of METTL14 increase the stability and amount of SRY-related HMG-box 4(SOX4) mRNA, activate the PI3K/Akt pathway, and change levels of N-cadherin, Vimentin, and E-cadherin. This shift promotes the EMT and enhances the metastatic capability of CRC cells (Fig. **3A**) [95]. Moreover, the m6A reader YTHDF1 plays a role in CRC by stabilizing and enhancing Rho/Rac guanine nucleotide exchange factor 2 mRNA expression through m6A binding, affecting the RhoA signaling pathway [96]. Additionally, research into the WNT pathway shows that its inhibitory gene, Axis inhibition protein 2(AXIN2), is regulated by ALKBH5. ALKBH5 removes m6A modifications from AXIN2 mRNA, which leads to its separation from the IGF2BP1 recognition protein and subsequent degradation. This activation of the Wnt/β-catenin pathway helps in the production of Dickkopf-related protein 1, which attracts myeloid-derived suppressor cells, thus promoting immunosuppression in CRC [97].

m1A methylation, an essential RNA modification, plays a significant role in the development and progression of CRC. ALKBH1, a key regulator of m1A, is notably overexpressed in CRC cells. The regulatory mechanisms of ALKBH1 include two main pathways: First, it reduces m1A modification on METTL3 mRNA, which increases METTL3 expression. This boost in METTL3 enhances the m6A modification on mothers against decapentaplegic (MAD)-related protein 7(SMAD7) mRNA, reducing its stability, decreasing SMAD7 protein synthesis, and promoting CRC cell metastasis [98]. Second, increased expression of CDC-like Kinase 3 (CLK3) enhances the invasiveness of CRC, usually regulated through autophagy or ubiquitination, with autophagy being the more dominant route. Meng Xue and colleagues found that CLK3 is also inhibited by Microfibril-associated glycoprotein 2(MFAP2), a process that can be reversed by the autophagy inhibitor 3-MA. This discovery shows that MFAP2 escalates CLK3 levels by blocking its autophagic degradation, thus influencing CRC's invasive capabilities. Additionally, ALKBH1 controls MFAP2 expression by increasing m1A levels on MFAP2 mRNA [99].

In the field of colorectal cancer, m5C RNA modification plays a regulatory role, influencing the disease's malignant characteristics. It regulates circRNA (circ_0102913), which is overexpressed in colorectal cancer, sequesters miR-571, and modifies the expression of Rac family small GTPase 2 (RAC2) mRNA. This interaction enhances tumor cell proliferation, migration, invasion, and tumor formation *in vivo* [100] (Fig. **3B**). Additionally, NSUN2 is known to control the proto-oncogene SKIL (also known as SnoN), a suppressor of transforming growth factor-β (TGFβ) signaling. NSUN2 promotes the growth of colorectal cancer cells by increasing m5C modification on SKIL mRNA and enhancing its interaction with YBX1, which stabilizes SKIL mRNA and increases Transcriptional co-activator with PDZ-binding motif (TAZ) expression [101].

Extensive research has shown that hypoxia and hypoxia-inducible factor (HIF)-1α are critical in the development and progression of CRC. The attachment of HIF-1α to the hypoxia response element (HRE) in the METTL1 mRNA promoter inhibits its transcription, leading to reduced METTL1 expression. This reduction lowers m7G modification on both mRNA and tRNA, with the effect on tRNA being significantly more pronounced. This difference suggests that m7G modification on tRNA may play a more vital role under hypoxic conditions and could be essential to the carcinogenic process [102] (Fig. **3C**).

Chi Jin *et al.* confirmed that both NAT10 and ac4C modification levels are significantly elevated in CRC. High NAT10 expression is associated with poor prognosis, lymph node metastasis, and distant metastasis in CRC. By binding to the 3′UTR region of kinesin family member 23(KIF23) mRNA, NAT10 increases ac4C modification and enhances the stability of KIF23 mRNA. This increase in protein levels activates the Wnt/β-catenin pathway, promoting CRC progression by inhibiting apoptosis and enhancing the proliferation, migration, and invasion of CRC cells, while arresting them in the G2/M phase [103] (Fig. **3D**). Additionally, NAT10 regulates the proliferation, migration, invasion, tumor formation, and metastasis of CRC cells by modulating the stability and protein translation of ferroptosis suppressor protein 1 mRNA [104].

### 3.4 RNA Modification and Liver Cancer

In 2022, liver cancer is projected to cause over 75,000 deaths globally, making it the third leading cause of cancer-related mortality after lung and colorectal cancers. Among males, liver cancer has the second highest mortality rate. In most regions, the incidence and mortality rates for men are two to three times higher than those for women. However, the primary risk factors vary significantly by region. In China and East Africa, chronic Hepatitis B virus(HBV) infection and exposure to aflatoxin are predominant risk factors, while in countries such as Egypt, Italy, and Japan, Hepatitis C virus(HCV) infection is the principal cause. Although the incidence of liver cancer has declined in East and Southeast Asia due to reduced prevalence of HBV and HCV and diminished exposure to aflatoxin, recent years have seen a gradual increase in incidence in low-risk countries due to increasing metabolic risk factors such as obesity, diabetes, non-alcoholic fatty liver disease, and alcohol consumption. Meanwhile, incidence rates remain high in regions with a high prevalence of risk factors [61]. Modifications of m1A, m6A, and m5C are intricately linked to the development of liver cancer, cholesterol synthesis, tumor cell proliferation, metastasis, invasion, and angiogenesis.

Gene set enrichment analysis has shown that RNA m1A modification is intricately associated with cell division, the MYC pathway, protein metabolism, and mitotic functions. This modification may influence the progression of hepatocellular carcinoma through the PI3K/Akt signaling pathway [105]. The m1A methyltransferase complex, composed of TRMT6 and TRMT61A, is highly expressed in advanced HCC and inversely correlated with patient survival rates. It activates the Hedgehog signaling pathway by elevating m1A modification levels on tRNA, which enhances the translation of Peroxisome proliferator-activated receptor delta (PPARδ) and stimulates cholesterol synthesis [68].

**Fig. (4):**
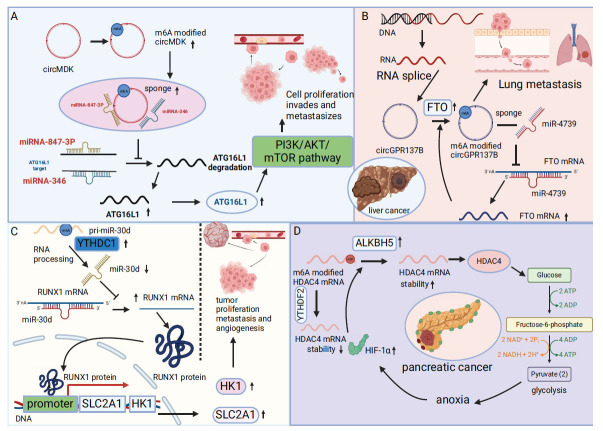
Depicts the role of m6A modifications in liver cancer and the role of m6A and m5C modifications in pancreatic cancer. (**A**) m6A modification increased the expression of circMDK, promoted the adsorption of miRNA-847-3P and miRNA-346, reduced the degradation of ATG16L1 mRNA by the two mirnas, and increased the expression of ATG16L1. Furthermore, the PIK/AKT/mTOR pathway promotes the proliferation, invasion and metastasis of cancer cells. (**B**) Promoting lung metastasis of liver cancer through circGPR137B/FTO/miRNA-4739 loop; That is, the up-regulation of FTO promoted the expression of circGPR137B, made it adsorb more miRNA-4739, reduced the degradation effect of miRNA-4739 on FTO mRNA, and caused the increase of FTO. The expression of circGPR137B is beneficial to lung metastasis of liver cancer. (**C**) YTHDC1 reduces the amount of miR-30d by recognizing m6A on pri-miR-30d, thereby inhibiting the degradation of RUNX1 mRNA by miR-30d and increasing its expression. RUNX1 promotes the gene expression of SLC2A1 and HK1 in the nucleus, causing tumor proliferation, metastasis and angiogenesis. (**D**) The upregulation of ALKBH5 increases the stability of HDAC4 mRNA by removing the m5C modification of HDAC4 mRNA (YTHDF2 recognizes the m5C modification of HDAC4 mRNA and reduces its stability) and promotes its expression. The high expression of HDAC4 can cause the enhancement of glycolysis of cancer cells and promote the hypoxia environment. ALKBH5 which was regulated by HIF-1α increased again.

METTL3, the principal RNA N6-adenosine methyltransferase, is significantly elevated in human hepatocellular carcinoma (HCC) and various solid tumors, correlating with adverse outcomes in patients with HCC. By overexpressing METTL3, the Suppressor of Cytokine Signaling 2(SOCS2) mRNA is targeted for recognition and degradation through YTHDF2, facilitating the progression and spread of HCC [106]. Additionally, circRNAs are gaining prominence in liver cancer research. For instance, Hsa_circ_0095868 (termed circMDK), derived from the fifth exon of the MDK gene, is an emerging oncogenic circRNA that is notably upregulated in HCC and closely linked to diminished survival rates among patients. circMDK sequesters miR-346 and miR-874-3p through its sponge-like mechanism, subsequently activating the PI3K/AKT/mTOR signaling pathway, enhancing cellular proliferation, migration, and invasion (Fig. **4A**) [107].

Furthermore, research has revealed that the aberrant expression of circGPR137B, which is substantially reduced in liver cancer, can significantly curtail cell proliferation, colony formation, and invasion. The presence of elevated levels of circGPR137B relies on FTO-mediated m6A modification and thwarts HCC tumorigenesis and metastasis through the circGPR137B/miR-4739/FTO regulatory feedback loop (Fig. **4B**) [108].

Wilms tumor-associated protein (WTAP), a critical element of m6A methylation, exhibits heightened expression in HCC tissues, correlating with unfavorable prognoses. It fosters the proliferation of hepatoma cells and tumor expansion both *in vitro* and *in vivo*. The proto-oncogene EST1 orchestrates the G2/M phase transition in HCC cells *via* a p21/p27-dependent mechanism. WTAP-mediated m6A modification can suppress EST1 translation, while Hu-Antigen R (HuR) stabilizes its RNA [109]. Yunhao Chen and colleagues posit that ALKBH5 hinders the proliferative and invasive capacities of HCC cells in laboratory and animal models. Its reduced expression in patients with HCC acts through post-transcriptional repression of LY6/PLAUR Domain Containing 1 (LYPD1), solidified by IGF2BP1 recognition, diminishing LYPD1 expression and enhancing proliferative and invasive tendencies in patients with HCC [110]. Conversely, some researchers argue that the m6A eraser ALKBH5, despite its high expression in HCC patients and association with poor outcomes, upregulates MAP3K8 expression by modulating m6A modification, thereby augmenting hepatoma cell proliferation and metastasis. Additionally, ALKBH5 promotes the activation of the JNK and ERK pathways, influences IL-8 expression, and fosters macrophage recruitment [111].

As an m6A reader, YTHDF2 is underexpressed in HCC and linked to a dismal prognosis, leading to reduced mRNA attenuation in IL11 and serpin family E members, triggering inflammation-driven tumorigenesis and vascular disruption [112].

The m5C modification, extensively studied in RNA alterations, holds significant sway in HCC. Notably, the ALYREF, upregulated in HCC, correlates with a higher Ki67+ cell rate, advanced TNM stage, and poorer prognosis. It boosts HCC cell proliferation, migration, invasion, and EMT by activating the STAT3 signaling pathway through direct interaction with the m5C modification site on the EGFR 3′ UTR, thereby stabilizing EGFR mRNA [113]. Despite their low abundance, lncRNAs such as H19 wield pivotal influence in various tumors, particularly HCC. H19 contributes to the poor differentiation of liver cancer, regulated by NSUN2-mediated m5C modification, and specifically interacts with the oncoprotein G3BP1 to foster MYC oncogene accumulation, further exacerbating low differentiation in HCC [114]. Furthermore, elevated NSUN2 expression in HCC correlates with several genes involved in critical signaling pathways such as Ras and PI3K-Akt, including Growth factor receptor-bound protein 2(GRB2), Ring Finger Protein 115 (RNF115), Apoptosis Antagonizing Transcription Factor (AATF), A Disintegrin And Metalloprotease 15 (ADAM15), Reticulon 3 (RTN3), and Hepatoma-Derived Growth Factor (HDGF) [115].

### 3.5 RNA Modification and Pancreatic Cancer

In 2022, an estimated 511,000 newly diagnosed cases of pancreatic cancer worldwide were reported, leading to 467,000 fatalities. This malignancy is notorious for its bleak prognosis, ranking as the sixth leading cause of cancer-related deaths globally and contributing to 5% of all cancer fatalities. Pancreatic cancer incidence is four times higher in nations with a high Human Development Index (HDI) compared to those with a low HDI, with notably elevated rates observed in Europe, North America, and Australia/New Zealand [61]. RNA modifications such as m1A, m6A, m5C, and ac4C play pivotal roles in the pathogenesis of pancreatic cancer, influencing various aspects of the disease including development, staging, cell cycle dynamics, metastasis, proliferation, invasion, drug resistance, and metabolic alterations.

Diminished expression of m1A and its regulatory enzyme ALKBH1 in pancreatic cancer correlates with a dire prognosis, expediting disease progression *via* the mTOR and Erythroblastic Leukemia Viral Oncogene Homologs (ErbB) signaling pathways [116]. Pancreatic cancer, acknowledged as a lethal solid tumor, is characterized by rampant cell proliferation and aggressive metastatic behavior. The m5C modification is closely linked to these traits of proliferation and metastasis. NSUN6 expression is associated with clinicopathological factors such as T stage and Ki67+ cell rate, with samples exhibiting low NSUN6 expression showing enrichment in cell cycle and G2M checkpoint, correlating with increased cell proliferation and tumor growth, thus suggesting NSUN6 as a potential marker for predicting tumor recurrence and patient survival. The m5C regulatory gene demonstrates significant associations with key genes involved in pancreatic cancer pathogenesis, including tumor protein p53, breast cancer 1, cyclin-dependent kinase inhibitor 2A, and ataxia telangiectasia mutated [117, 118].

NAT10 functions as an oncogene with elevated mRNA and protein levels in pancreatic ductal adenocarcinoma (PDAC) tissues. Heightened NAT10 protein expression in patients with PDAC is closely associated with poor outcomes. NAT10 enhances the stability of tyrosine kinase AXL mRNA through ac4C acetylation, leading to increased AXL expression, thus promoting the proliferation and metastasis of PDAC cells and contributing to its oncogenic properties [119].

In the process of m6A modification, pivotal regulatory factors such as YTHDF2, METTL14, ALKBH5, METTL3, YTHDC1, and IGF2BP2 play crucial roles. The absence of ALKBH5 significantly influences the detrimental clinicopathological characteristics observed in patients with pancreatic cancer. Increased expression of ALKBH5 restrains tumor proliferation, migration, and invasion, and inhibits tumor growth *in vivo*, a process intricately linked to period circadian regulator 1(PER1). The expression levels of ALKBH5 and PER1 typically fluctuate concurrently, with AlkBH5 expression stability maintained through the removal of m6A modification on PER1 mRNA. The impact of m6A modification on PER1 mRNA degradation depends on recognition and binding by YTHDF2. PER1 combats pancreatic cancer by activating the ATM signaling pathway [120]. Simultaneously, chemotherapy resistance in pancreatic cancer, contributing to its classification as one of the most lethal solid tumors, is closely linked to hepatic function. ALKBH5 also contributes to drug resistance in pancreatic cancer by modulating mRNA expression through its influence on m6A modification of long non-coding RNA (DDIT4-AS1). m6A readers HuR and IGF2BP1 are known for stabilizing RNA, with RIP experiments confirming HuR's ability to recognize m6A modification on DDIT4-AS1 and enhance its expression. DDIT4-AS1b signifies a grim prognosis in pancreatic cancer, influencing stem cell traits and response to chemotherapy *via* activation of the mTOR signaling pathway. Additionally, phosphorylated up-frameshift 1 degrades DNA damage-inducible transcript 4 mRNA, a process implicated in tumor stem cell function and resistance to chemotherapy [121]. METTL14 and p53 apoptosis effectors related to PMP-22 (PERP) mRNA are crucial in pancreatic cancer proliferation and metastasis, especially PERP mRNA, whose turnover accelerates post m6A modification, resulting in reduced mRNA and protein expression, thus promoting pancreatic cancer growth and spread [122]. METTL14 expression is significant in pancreatic cancer and collaborates with METTL3 to enhance Inhibitingthe inhibition of DNA binding 2 mRNA stability through m6A modification. This stabilization depends on YTHDF2 recognition and binding, influencing the expression of stem cell markers NANOG and SOX2 through activation of the PI3K-AKT signaling pathway [123]. The aggressive progression of pancreatic cancer is closely associated with aerobic glycolysis, with microRNA (miR-30d) pivotal in regulating this metabolic pathway. Furthermore, miR-30d exerts regulatory control over tumor proliferation, migration, and angiogenesis, with its overexpression serving to hinder tumor growth. The orchestration of aerobic glycolysis regulation involves transcription factors, wherein miR-30d modulates the expression of Solute carrier family 2 member 1(SLC2A1) and Hexokinase 1(HK1) *via* the transcription factor RUNX1, thereby restraining tumor progression. YTHDC1 plays a pivotal role in recognizing pre-miR-30d and facilitating its degradation, consequently reducing miR-30d levels [124] (Fig. **4C**). The m6A reader protein IGF2BP2 boosts the expression of B3GNT6 mRNA by modulating its stability. Co-expression of IGF2BP2 in patients with pancreatic cancer exacerbates tumor proliferation and migration, thereby hastening disease progression. B3GNT6, an enzyme belonging to the glycosyltransferase family, is implicated in these processes [125]. Pancreatic cancer is notorious for its hypoxic environment among solid tumors, with dynamic shifts in m6A modifications aiding tumor cells in adapting to hypoxia. Under such conditions, the upregulation of HIF1 enhances the expression of ALKBH5, leading to increased m6A modification on histone deacetylase 4(HDAC4) mRNA. This modified mRNA is recognized and stabilized by YTHDF2, thereby augmenting the glycolytic capacity and metastatic potential of pancreatic cancer cells [126] (Fig. **4D**).

## 4 RNA MODIFICATION AND THERAPY

In recent years, research into RNA modification has revealed its crucial role in cancer therapy. The genesis, progression, and treatment resistance of tumors are intricately linked to factors such as oncogene activation, DNA damage repair mechanisms, the presence of cancer stem cells, alterations in the tumor microenvironment, induction of hypoxic states, metabolic changes, and the initiation of autophagy. Studies indicate that various RNA modifications can therapeutically influence the stability of specific gene transcripts or signaling pathways. For instance, in chemotherapy, cisplatin targets the MAPK signaling pathway and the GLS1 gene to treat pancreatic and colorectal cancers, respectively. Gemcitabine addresses pancreatic cancer by impacting WIF-1, the Wnt signaling pathway inhibitor 1, PHD finger protein 10, and cytidine deaminase (CDA). It combats colorectal cancer by affecting the Wnt pathway, c-myc, and miR-181d-5p. Sorafenib, a targeted therapy for liver cancer, modulates METTL3, which subsequently affects FOXO3's role in autophagy. Entacapone impedes gluconeogenesis in liver cancer cells by competing with FTO for binding sites [127] Concurrently, the m6A modification regulator also plays a part in these drug actions [128]. The immunotherapy drug PD-1 targets the IFN-γ-Stat1-Irf1 pathway in colorectal cancer treatment, a process closely associated with RNA m5C modification. Knocking out YBX1, an m5C modification reader can reverse chemotherapy resistance by inhibiting PD-L1 expression and activating T cells within the tumor microenvironment [59]. Additionally, resistance to the tyrosine kinase inhibitor lapatinib, a primary treatment for liver cancer, is increasing. However, this can be mitigated by reducing the components of the tRNA m7G methyltransferase complex, specifically METTL1 and WDR4. Targeted therapy involving METTL1 is also being explored as a novel strategy for treating liver cancer [129]. NAT10 plays a role in the resistance to gefitinib in treating esophageal cancer. Depleting NAT10 in conjunction with gefitinib can synergistically hinder the progression of esophageal cancer [72]. Furthermore, M1A-related regulatory factors, including the m1A writers TRMT6 and TRMT61A, and the eraser FTO, have been employed in disease treatment. Thiram effectively curtails the self-renewal and growth of liver cancer cells by disrupting the interaction between TRMT6 and TRMT61A [130].

## CONCLUSION

This review provides a comprehensive overview of RNA modifications, including m6A, m5C, m1A, m7G, and ac4C, highlighting the fundamental roles of their regulatory factors. It explores the characteristics of these modifications and their implications in various diseases, with a specific focus on their impact on digestive tract tumors. These modifications influence growth, proliferation, migration, and immune evasion in esophageal cancer. They are implicated in metastasis, invasion, and proliferation in gastric cancer. In colorectal cancer, they facilitate tumor growth, migration, angiogenesis, and immune suppression. In liver cancer, they are intricately linked to onset, progression, cholesterol synthesis, tumor cell growth, metastasis, invasion, and angiogenesis. In pancreatic cancer, they play crucial roles in development, staging, cell cycle regulation, migration, proliferation, invasion, drug resistance, and metabolic alterations. Moreover, they significantly affect the prognosis of cancer patients. These insights offer a profound understanding and novel perspectives for the diagnosis and treatment of digestive tract tumors, thereby enhancing society's ability to manage these conditions more effectively.

The inherent limitations of RNA modification detection technology hinder the thorough exploration of various modification impacts and restrict comprehensive analysis of RNA modifications under normal physiological conditions. This impedes holistic analysis and management of the onset and progression of various diseases, stifling innovation in diagnostic and therapeutic strategies. As research into the role of individual modifiers in disease progresses, it becomes increasingly apparent that the effects of multiple modifiers are more intricately linked to physiological states, rendering them more suitable for targeted medication development. With advancements in drug development technology, combination therapies have emerged as a predominant trend. Consequently, combined or collaborative therapies targeting multiple RNA modifications may represent a significant future research avenue.

## Figures and Tables

**Table 1:** Describes the effects of m6A, m1A, m5C, m7G, and ac4C modifications on mRNA, tRNA, and rRNA, as well as their respective regulatory factors and their modification sites.

-	**Regulator**	**Structural Characteristics**	**Position**	**Function**	**References**
m6A	Writer: METTL3, METTL14 Reader:YTHDC1, YTHDF1, YTHDF2, YTHDF3, eIF3, METTL3, HNRNPC, HNRNPG, IGF2BP1, IGF2BP2, IGF2BP3, FMRP; Eraser: FTO, ALKBH5	IGF2BP family:two (RRMs) and four KH domains. KH domain:(binding to RNA), RRM domain:(binding stability); hnRNPA2B1:two RNA recognition motifs (RRMs) and glycine-rich low complexity region (LC)	3 'untranslated regions (UTRs) and conserved locations near the stop codon	mRNA: Destabilizing effect; Regulates translation, splicing , inflammatory pathways and oncogene expression, signal transduction, and gene expression.	[1, 2] [4, 9] [18, 11] [23, 24] [26, 28] [31, 32]
	Writer: ZCCHC4, METTL5	-	Adenosine site 1832 for 18S rRNA and adenosine site 4220 for 28S rRNA	rRNA: Increase ribosome heterogeneity and regulate translation .	[1, 33]
	Writer:METTL3; YTHDC1; KIAA129; METTL14; WTAP; IGF2BP2 Reader:YTHDF3, eIF4G2 ;YAP1;YTHDF1 Eraser:ALKBH5	-	-	circRNA: Promotes tumorigenesis and gene silencing	[1, 34]
m1A	Writer: TRMT6, TRMT61A, TRMT61B, TRMT10C, NML Reader: YTHDF1, YTHDF2, YTHDF3, YTHDC1 Eraser: ALKBH1, ALKBH3, ALKBH7, FTO	TRMT6 - TRMT61A complex: TRMT61A (catalytic subunit), TRMT6 (recognition subunit) ;ALKBH3:β - hairpin (β4-loop-β5, β ' -loop-β ' ')and α 2 spirals; YTH family: The bubble-like area around Asp194 and residues (Thr133)	5 'untranslated region (5' UTR), encoding sequence (CDS), or 3 'untranslated region (3' UTR)	mRNA: Regulation of translation and protein synthesis (forming T-Loop or hairpin structures); Highly structured regions around the start codon are correlated; Influences RNA secondary structure formation, cell cycle, signaling pathways, cell proliferation, migration capacity, cell necrosis, Glucose metabolism .	[36, 39] [40, 41] [42, 43] [44]
	Writer: TRMT6/ TRMT61A, TRMT10C/ SDR5C1, TRMT61B Eraser: ALKBH1 ALKBH3, FTO	ditto	Digits 9, 14, 22, 57 and 58	tRNA: Structural stability, folding, mitochondrial respiration, Related to structural thermal stability; Retrofidelity and efficiency; Processing of precursor RNA; translation and translation efficiency	[48, 49]
m5C	Writer: NSUN2, NSUN4, NSUN6, DNMT2 Reader: YBX1, Aly/REF Output factor (ALYREF) RAD52, LIN28B, FMRP Eraser: ALKBH1, TET1	NSUN protein: RNA recognition motif (RRM) and Rossman-fold catalytic core that accommodates SAM cofactors	5 'UTR, CDS, 3' UTR and translation start position	mRNA: mRNA dependent recombination, stable mRNA, translation, promote nuclear output, fat formation, glycolysis, exosome secretion, tumor malignancy and drug resistance, signaling pathways; cell proliferation, invasion and cell cycle	[50, 51]
	Writer: DNMT2, NSUN2/3/6, TET2, Eraser: ALKBH1	-	C72 and C38 sites of tRNA; C34, C40, C48, C49, and C50 on certain Rnas	tRNA: Stress response, translation efficiency and accuracy; Codon recognition; Levitt pair “hinteractions and tRNA tertiary structure; Mitochondrial respiratory function and protein translation	[50, 51]
	Writer: NSUN1/4/5 Reader: YTHDF2	-	28S/25S rRNA3761 and human 4413	rRNA: Bacterial drug resistance; Structural stability of rna-trna-mrna; Translation and mitochondrial ribosome assembly; Cytoplasmic translation; Stress resistance and life extension; Folding of rRNA in ribosomes	[50, 51]
m7G	Writer: RNMT/RAM, METTL1/ WDR4, WBSCR22/ TRMT112 Reader: eIF4E family (eIF4E1/eIF4E, eIF4E2/4EHP and eIF4E3), Ago2	RNMT/RAM methyltransferase complex methyl donor: SAM	5 'UTR, coding sequence (CDS) and 3' UTR are concentrated in AG enriched regions	mRNA: The cap structure of mRNA 5 'UTR was regulated. Mediates mRNA nuclear export and translation process; Reduce the translation efficiency of mrna; Protect RNA from exonuclease cutting, affecting RNA processing	[48, 54]
	Writer:METTL1/ WDR4	-	Variable ring 46^th^; AG sequence; tRNA anti-codon region	tRNA: Stabilizes and influences the tertiary structure and function of tRNA; Participate in tRNA attenuation; Influence the effectiveness of mRNA translation; The increase of ribosome suspension at tRNA binding site (site A) hindered ribosome translocation. Promote the reaction rate of other tRNA modifying enzymes; Promote cell cycle; Improve the translation efficiency of oncogenes	[54]
	Writer:WBSCR22/ TRMT112	WBSCR22 / TRMT112 complex, TRMT112: cofactor, catalytic subunit: WBSCR22	The G1575 site of yeast 18S rRNA; G1639 site of human rRNA; The G1405 site of bacterial 16S rRNA	rRNA: Associated with bacterial resistance to aminoglycosides	[54]
ac4C	Writer: NAT10	NAT10: N-acetyltransferase domain, ATP/GTP binding domain and ATPase domain	5 'UTR, coding sequence (CDS)	mRNA: regulation of translation; Increase mRNA half-life	[55, 56]
	Writer: NAT10	ditto	Position 1773 in the yeast 18S rRNA;position 4 of the terminal helix in the rat 18S rRNA	rRNA: Affects the translation fidelity of RNA or regulates the assembly process of ribosomes; Influence cell growth rate and rRNA stability; Provide binding sites for t RNA, improve the ability of t RNA to correctly recognize codons, and facilitate the formation of peptide chains	[56]
	Writer: NAT10	ditto	-	tRNA: Stabilizes the internal conformation of ribose C3 ', thereby enhancing GC-base interactions and facilitating correct codon reading during translation.	[56]

**Table 2:** Describes the regulatory effects and regulatory pathways of m6A, m1A, m5C, m7G, and ac4C modifications in digestive tract tumors (including esophageal cancer, gastric cancer, colorectal cancer, liver cancer, and pancreatic cancer) respectively.

**Tumor**	**Modification**	**Regulators**	**Function**	**Regulatory Pathway**	**References**
ESCA	m7G	METTL1/ WDR4	ESCC progression	PRTOR/ULK1/ autophagy axis;METTL1/MTORC1/ULK1/ autophagy axis;	[58, 59]
	ac4C	NAT10	ESCA tumorigeness and progression	-	[68]
	m6A	METTL3/ YTHDF2; IGF2BP1; SHMT2; FTO	Cell proliferation and tumour growth;metastasis; boosting EC tumorigenesis	β-catenin, cyclin D1, c-Myc and PKM2.; p38 MAPK pathway; METTL3/FTO/ALKBH5/IGF2BP2-dependent way(c-myc); WNT; Notch signaling pathway; FTO/LINC00022 axis;METTL3/IFIT2	[60-66]
	m5C	NSUN2	Tumorigenicity and progression	PI3K/AKT and ERK/MAPK signaling (NSUN2-GRB2 axis)	[67]
GC	m6A	ALKBH5/ IGF2BP3; METTL3; METTL14 YTHDF1; FTO; IGF2BP2; WTAP;	Promoted invasion and migration; promoted tumour growth and liver metastasis;promoted lymph node metastasis capabilities; epithelial-mesenchymal transition (EMT); suppresses gastric cancer progression;drug resistance	ALKBH5/ PKMYT1 axis;H3K27 acetylation/METTL3/HDGF mRNA axis; c-Jun/ c- Fos/SREBP1 axis ; METTL3/ ZMYM1/E-cadherin signaling ; METTL3-PBX1-GCH1-BH 4(tetrahydrobiopterin) axis ; METTL14-circORC5-miR-30c-2-3p/AKT1S1 /EIF4B axis; YTHDF1- frizzled7 (FZD7)-Wnt/b-catenin pathway; IGF2BP3/OIP5-AS1/hnRNPA1 axis (PKM2 signaling pathway); FTO-caveolin-1.; IGF2BP2- CSF2-reprogramming Mesenchymal stem cells (MSCs);AGAP2-AS1/WTAP/STAT3 pathways;IGF2BP2-IGF1R-RhoA-ROCK signaling pathway	[69-80]
	m5C	NSUN2/ YBX1	Peritoneal metastasis and colonization ; promoted migration, invasion, and Neural invasion (NI) potential;	NSUN2-ORAI2(AMPK pathway); DIAPH2-AS1-NSUN2-NTN1 axis; FOXC2-AS1-NSUN2-FOXC2	[81-83]
	ac4C	NAT10	Promoted cellular G2/M phase progression, proliferation and tumorigenicity; enhances hypoxia tolerance; promoted the metastasis	NAT10 -MDM2-p53 aixs; NAT10/SEPT9/HIF-1α positive feedback loop ;NAT10-SMYD2-neutrophil extracellular traps (NETs)	[84-86]
CRC	m1A	ALKBH1	Migration and invasion; lymph node metastasis and distant metastasis	ALKBH1- METTL3-SMAD7; MFAP2-CLK3 signaling axis	[94, 95]
	m6A	METTL3; IGF2BP2/3YTHDF3;METTL14/YTHDF2; YTHDF1;ALKBH5	Promote vasculogenic mimicry (VM); facilitates tumor metastasis; tumorigenesis ; drives immune suppression	m 6A-GLUT1-mTORC1 axis ; m6A-IGF2BP2/3-EphA2 and VEGFA(PI3K/AKT/mTOR and ERK1/2 signaling); LINC00460-(METTL3)/(IGF2BP2 and DHX9)-HMGA1; lncRNA GAS5-YAP-YTHDF3 axis;METTL14/YTHDF2-SOX4-mediated EMT process and PI3K/Akt signals;YTHDF1-m 6A-ARHGEF2; ALKBH5-N6-methyladenosine-AXIN2-Wnt-DKK1 axis	[87-93]
	m5C	NSUN5; circSUN2/ IGF2BP2; NSUN2/ YBX1	Augments malignant properties; promotion of EMT; promoted colorectal cancer cell growth	m5C-Circ_ 0102913-miR-571-RAC2 axis; NSUN2-Y-box binding protein 1 (YBX1)-SKIL-PAZ	[96, 97]
	m7G	METTL1	Development of colorectal cancer	METTL1-HIF-1α	[98]
	ac4C	NAT10	CRC progression	NAT10/KIF23/GSK-3β/β-catenin axis ; NAT10-ferroptosis suppressor protein 1 (FSP1)	[99, 100]
HCC	m1A	TRMT6/ TRMT61A	Self-renewal and tumourigenesis	PI3K/Akt and MYC signaling; m1A-peroxisome proliferatoractivated receptor-delta (PPARδ)-cholesterol synthesis-Hedgehog signaling	[101, 102]
	m6A	METTL3/ YTHDF2; FTO ; METTL14 IGF2BP1;WTAP; FTO;ALKBH5	Cell cycle arrest and suppresses the proliferation ; regulate cell proliferation, migration and invasion; modulated the G2/M phase ;promote PD-L1+macrophage recruitment; provoked inflammation, vascular reconstruction	FTO-pyruvate kinase M2 (PKM2) ; circMDK-IGF2BP1-miR-346/874-3p-ATG16L1 (Autophagy Related 16 Like 1)axis(PI3K/AKT/mTOR signaling pathway); HuR-ETS1-p21/p27 axis; circGPR137B/miR-4739/FTO axis; ALKBH5-MAP3K8-JNK and ERK pathways; hypoxia-inducible factor-2α (HIF-2α)-YTHDF2 - interleukin 11 (IL11) and serpin family E member 2 (SERPINE2) mRNAs; ALKBH5/LYPD1 axis	[103-109]
	m5C	ALYREF; NSUN2	Cell proliferation, migration, invasion, epithelial-mesenchymal transition (EMT) and metastasis	ALYREF-EGFR mRNA-STAT3 signaling pathway; NSUN2-H19 RNA-G3BP1-MYC accumulation; PI3K-Akt, ErbB, and Ras signaling pathway	[110-112]
PAAD	m1A	ALKBH1	-	mTOR and ErbB signaling pathway	[113]
	m5C	NSUN6; NSUN2	Cell proliferation like cell cycle and G2M checkpoint ; DNA repair, epithelial differentiation and tumor stromal interactions	-	[114, 115]
	ac4C	NAT10	Cell proliferation and metastasis;	NAT10-receptor tyrosine kinase AXL;	[116]
	m6A	YTHDF2; METTL14 ALKBH5; METTL3 YTHDC1; IGF2BP2;	Reduced tumoural proliferative, migrative, invasive; stemness and suppressed chemosensitivity to GEM; stemness maintenance; suppressing aerobic glycolysis. promoted glycolytic metabolism	m6A-YTHDF2-PER1(ATM-CHK2-P53/CDC25C signalling); CLK1/SRSF5 pathway ; m6A-DDIT4-AS1-DDIT4(mTOR pathway); m6A-PERP mRNA; m6a-YTHDF2-ID2 mRNA-NANOG and SOX2 (PI3K-AKT pathway); YTHDC1-miR-30d-RUNX1-SLC2A1 and HK1; IGF2BP2-B3GNT6 mRNA; ALKBH5/HDAC4/HIF1α positive feedback loop	[117-123]

## LIST OF ABBREVIATIONS

AATF: Apoptosis Antagonizing Transcription Factor
ABH1: ATP-binding Cassette Subfamily H Member 1
ac4C: N4-acetylcytosine
ADAM15: A Disintegrin And Metallopr
AGAP2-AS1: Arf GTPase Activating Protein 2 Antisense RNA 1
AKT: Protein Kinase B
AKT1S1: Akt Substrate 1S1
ALKBH5: AlkB Homolog 5
ALYREF: Aly/REF Export Factor
APC: Adenomatous Polyposis Coli
AXIN2: Axis Inhibition Protein 2
BH4: Tetrahydrobiopterin
CBC: Cap-binding Complex
CDA: Cytidine Deaminase
CDS: Coding Sequence
CLK3: CDC-like Kinase 3
c-Myc: Vian Myelocytomatosis Virus Oncogene Cellular Homolog
CoREST: Corepressor for Element-1-silencing Transcription Factor
CSF2: Colony-stimulating Factor 2
CtBP: E-cadherin Promoter *via* the C-terminal Binding Protein
cyclin D1: G1/S-specific Cyclin-D1
DNMT2: DNA Methyltransferase 2
EGFR: Epidermal Growth Factor Receptor
eIF3: Eukaryotic Initiation Factor 3
EIF4B: Eukaryotic Translation Initiation Factor 4B
EMT: Epithelial-mesenchymal Transition
ErbB: Erythroblastic Leukemia Viral Oncogene Homologs
ERK: Extracellular Signal-regulated Kinase
ESCC: Esophageal Squamous Cell Carcinoma
FMRP: Fragile X Mental Retardation Protein
FOXC2-AS1: Forkhead Box C2 Antisense RNA 1
FOXO3: Forkhead Box O_3_
FTO: Fat Mass and Obesity-associated protein
FZD7: Frizzled7
GAS5: Growth Arrest Specific 5
GRB2: Growth Factor Receptor-bound Protein 2
HBV: Hepatitis B Virus
HCV: Hepatitis C Virus
HDAC4: Histone Deacetylase 4
HDGF: Hepatoma-Derived Growth Factor
HIF-1: Hypoxia Inducible Factor-1
HIV-1: Immunodeficiency Virus type 1
HK1: Hexokinase 1
HNRNP: Heterogeneous Nuclear Ribonucleoprotein
hnRNPA1: Heterogeneous Nuclear Nucleoprotein A1
HRE: Hypoxia Response Element
HuR: Hu-Antigen R
IFIT2: Interferon-induced Protein with Tetratricopeptide Repeats 2
IGF2BP: Insulin-like Growth Factor 2 mRNA Binding Protein
IL6: Interleukin 6
KIAA129: Kinase Interacting with the Armadillo Repeat-containing Protein 1299
KIF23: Kinesin Family Member 23
LIN28B: Lin-28 Homolog B
LSD1: lysine-specific Demethylase 1
LYPD1: LY6/PLAUR Domain Containing 1
m1A: 1-methyladenosine
m5C: 5-methylcytosine
m6A: N6-methyladenosine
m7G: 7-methylguanosine
MAPK14: Mitogen-activated Protein Kinase 14
MDM2: Mouse Double Minute 2
MeRIP-seq: Methylated RNA Immunoprecipitation Sequencing
METTL: Methyltransferase Like
MFAP2: Microfibril-associated Glycoprotein 2
MKK6: Mitogen-activated Protein Kinase Kinase 6
MSCs: Mesenchymal Stem Cells
NADH: Nicotinamide Adenine Dinucleotide Hydogen
NAT10: N-acetyltransferase 10
ND5: NADH Dehydrogenase Subunit 5
NETs: Neutrophil Extracellular Traps
NI: Neurological Invasion
NMD: mRNA Decay
NML: Nuclear Myosin 1-Like Protein
Npl3: Nucleolar Protein Like 3
NSUN2/4/6: NOP2/Sun RNA Methyltransferase 2/4/6
OIP5-AS1: Opioid Induced Protein 5 Antisense RNA 1
ORAI2: ORAI Calcium Release-Activated Calcium Modulator 2
PDAC: Pancreatic Ductal Adenocarcinoma
PER1: Period Circadian Regulator 1
PERP: p53 Apoptosis Effector Related to PMP-22
PI3K: Phosphoinositide 3-kinase
PKM2: Pyruvate Kinase Isozymes M2
PKMYT1: Protein Kinase, Membrane-associated Tyrosine/Threonine 1
PPARδ: Peroxisome Proliferator-activated Receptor Delta
RAC2: Rac Family Small GTPase 2
RAD52: RAD52 DNA Recombination and Repair Protein
RAM: RNA guanine-7 Metyltransferase-associated Protein
Rcm1: Replication Component 1
RFM: Resting Metabolic Rate Fat-Free Mass
RGG: Arg-Gly-Gly
RNF115: Ring Finger Protein 115
RNMT: RNA Guanine-7 Methyltransferase
RPRD1B: Regulation of Nuclear Pre-mRNA Domain Containing 1B
RPTOR: Regulatory Associated Protein of mTOR Complex 1
RRM: RNA Recognition Motif
RTN3: Reticulon 3
SAM: S-adenosyl-L-methionine
SDR5C1: Short-Chain Dehydrogenase/Reductase Family 5C Member 1
SHMT2: Study Highlights the Involvement of Serine Hydroxymethyltransferase 2
SLC2A1: Solute Carrier Family 2 Member 1
SMAD7: Mothers against Decapentaplegic MAD)-related Protein 7
SMYD2: Set and MYND Domain Containing 2
SOCS2: Suppressor Of Cytokine Signaling 2
SOX4: SRY-related HMG-box 4
SPOUT: SpoU rRNA Methylase Family
STAT3: Signal Transducer and Activator of Transcription 3
TAZ: PDZ-binding Motif
TET1: Ten Eleven Translocation Methylcytosine Dioxygenase 1
TGFβ: Transforming Growth Factor-β
TRIM21: Tripartite Motif-containing Protein 21
TRMT6/61A: tRNA Methyltransferase 6/61A
ULK1: unc-51 Like Autophagy Activating Kinase 1
WBSCR22: Williams-Beuren Syndrome Chromosome Region 22
WDR4: WD Repeat-containing Protein 4
WTAP: Wilms Tumor 1 Associated Protein
YAP1: Yes Associated Protein 1
YBX1: Y-Box Binding Protein 1
Yral: RNA Binding Protein Yra1
YTHDC: YTH Domain-containing Protein
YTHDF: YTH Domain-containing Family Protein
ZCCHC4: Zinc Finger CCHC Domain-containing Protein 4
ZMYM1: Zinc Finger MYM-type Containing 1
